# Nutrients patterns and attention deficit hyperactivity disorder among Egyptian children: a sibling and community matched case-control study

**DOI:** 10.1038/s41430-023-01345-0

**Published:** 2023-09-26

**Authors:** Samia Dahshan Gumma, Sally Fawzy Elotla, Omneya Youssef Ibrahim, Nadia Hosny Rizkalla

**Affiliations:** 1 Department of Public Health, Occupational and Environmental Medicine, Faculty of Medicine, Suez Canal University, Ismailia, Egypt; 2 Department of Neuropsychiatry, Faculty of Medicine, Suez Canal University, Ismailia, Egypt

**Keywords:** Risk factors, Epidemiology

## Abstract

**Background:**

Attention Deficit Hyperactive Disorder (ADHD) diagnosis has been growing among children, with great public health concern. The relationship between nutrient intake pattern and ADHD remains unclear.

**Aim:**

To identify the nutrient intake patterns and its association with ADHD in children.

**Subjects and methods:**

The study involved 146 children with ADHD, 141 control sibling, and 146 community controls. ADHD diagnosis was confirmed using the Diagnostic and Statistical Manual of Mental Disorders, 5th Edition (DSM-V) criteria following the assessment by the long-form Conner’s Scale. Dietary intakes were assessed using a semi-quantitative food frequency questionnaire. Nutrients patterns were identified using the principal component analysis (PCA).

**Results:**

ADHD children have significantly higher mean total energy intake than community controls and siblings (8867.9, 4481.9 and 7308.2 KJ, respectively, *p* < 0.001). Four nutrient patterns extracted by the PCA explained 75.9% of the total variance. Lower tertiles of “predominantly calcium-phosphorus; pattern 1” and “predominantly-vitamins; pattern 3” were significantly associated with increasing odds of ADHD, compared to community controls (*p* for trend: 0.002 and 0.005, respectively), while the same associations were noted in “predominantly-vitamins” and “predominantly Zinc-Iron; pattern 2” when compared to siblings (*p* for trend: <0.001 and <0.001, respectively). However, Higher tertiles of macronutrients; pattern 4” were associated with increased ADHD odds, compared to either community controls or siblings (*p* for trend: 0.017 and <0.001, respectively).

**Conclusion:**

Lower intakes of nutrients patterns of minerals and vitamins, and higher intakes of macronutrients were associated with increased likelihood of ADHD in children.

## Introduction

Children are highly vulnerable to unhealthy diets which adversely affect their body development and make them susceptible to many morbidities through life course [1]. Attention Deficit Hyperactive Disorder (ADHD) is one of the most diagnosed childhood neurodevelopmental disorders that continue into adulthood [2]. Children with ADHD are characterized by developmentally inappropriate levels of inattention and hyperactivity, and commonly presented with either predominantly inattentive, predominantly hyperactive-impulsive, or combined presentations [3].

Because of its rising global prevalence and burden, ADHD has become an alarming global public health issue. Globally, the prevalence of childhood ADHD has increased from 7.2% in 2015 to 13.3% in 2021 [4, 5]. Estimates of ADHD prevalence among children are almost consistent across Arab and non-Arab countries (9.4%, 8.5%, 9.5% and 5–12% in United States, Korea, and Jordan, respectively) [6]. In Egypt, the prevalence ranged from 9.4% to 21.8% [7–9]. ADHD during childhood is associated with impaired school performance due to learning disorders, impaired socialization, and occasionally conduct disorders and bipolar manifestations [10, 11].

The exact etiology of ADHD is still unknown. However, the interaction between biological and environmental factors could explain the rising incidence of ADHD [12, 13]. Many studies claimed nutritional deficiencies (e.g., zinc, magnesium, ferritin, and omega-3 fatty acid) for the development of ADHD [14–20].

Recent evidence has supported the hypothesis that not only do specific nutrient intake variations affect ADHD occurrence but also the entire diet does [21]. Higher intake of high-fat, high-sugar, and high-additives diets have been significantly associated with ADHD [22]. Although many studies have investigated the association between dietary intake or pattern and ADHD [19, 23, 24], studies about the nutrient patterns in ADHD children is scarce. Therefore, this study aimed at identifying the nutrients patterns and its association with ADHD in children.

## Subjects and methods

This is a matched 1:2 case-control study of school children aged 6–10 years old, conducted in the child psychiatry clinics at Suez Canal University and Suez Canal Authority hospitals in Ismailia city, Egypt from February 2019 to the end of December 2019. The study proposal was approved by the ethics committee for medical research at Faculty of Medicine, Suez Canal University, Egypt (Ethical approval number: 3350). Informed consent was obtained from the guardian of all children who participated in this study.

Cases included newly diagnosed ADHD children. Parents of recruited children were first interviewed with Arabic version of the Long-Form Conner’s Scale, then the scale’s responses were assessed by a psychiatrist to confirm ADHD diagnosis according to the Diagnostic and Statistical Manual of Mental Disorders, 5th Edition (DSM-V) criteria for ADHD in children [25]. DSM-V criteria for ADHD emphasizes a persistent pattern of inattention and/or hyperactivity–impulsivity that interferes with functioning or development. Children up to age 16 years should have six or more symptoms of inattention and/or hyperactivity–impulsivity for at least 6 months, not better explained by another mental disorder (such as a mood disorder, anxiety disorder, dissociative disorder, or a personality disorder), and to an extent that is disruptive and inappropriate for developmental level [25].

Two control groups were identified: a community control group (I), and sibling cases control group (II). Cases and controls were matched for sex and age (6–45-month group difference). Children in either control group were only eligible if they were negative for ADHD using the same evaluation procedures and diagnostic criteria for cases group. Children who had a medical history of neurological or psychiatric conditions and those who were on regular medications for at least 3 months that could affect their nutritional intake (e.g., anti-histaminic, beta-blockers, antipsychotics, and hormonal therapy) were excluded [26].

A sample size of 132 children in each group was calculated using Epi-Info 6.04 software at 95% level of confidence, 80% study power, and a medium effect size of 0.4, given a 0.2 probability of sweet pattern among controls, and an odds ratio 2.2 between the case group and control groups. The number per group was increased to 146 to compensate for a 10% dropout rate.

All recruited children’s caregivers were interviewed for dietary assessment using a semi-quantitative food frequency questionnaire (FFQ) [27]. The FFQ comprised 137 food items that commonly consumed by Egyptian children, according to the Egyptian Food Composition Table [28]. Caregivers were asked to report on their children’s average frequency of consumption over the past year, using a scale of nine frequency levels ranging from “almost never” to “6-times per day”. The FFQ was first reviewed for its content by a panel of three experts, then tested for internal consistency and test-retest reliability using a pilot study of 43 children (interviewed twice, 2-weeks a part). Pilot testing revealed acceptable internal consistency (Cronbach alpha = 0.81) and test-retest reliability (Pearson’s correlation coefficient = 0.85). Caregivers were also asked to indicate the consumed portion sizes per single occasion of the selected 116 food items, guided by a portion size atlas. The portion size atlas was prepared by the authors and comprised culturally adapted photos of food items in various sizes using standard portion size estimation tools (e.g., tennis ball and deck cards) and commonly used household measures. The atlas also contained the weight estimates (in grams) of each portion size. Then, the average daily consumption of nutrients and total energy were calculated given the food frequencies and portion sizes and guided by the Egyptian Food Composition Table [28].

Height (cm) and body weight (kg) were measured for all recruited children, while we used WHO Anthro software (version 3.2.2, January 2011) to calculate children’s BMI percentile given the standardized age- and gender-specific curves [29].

All statistical analyses were performed using Statistical Package for Social Science (SPSS) version 23. Quantitative data were described as mean (SD) or median, while categorical data were summarized as frequencies and percentages. The daily nutrient intake was expressed in energy-adjusted values. Energy adjustment was performed using the nutrient residual method, in which, energy-adjusted nutrient intake equals the sum of (a) the residual from a regression model with nutrient intake as the dependent variable and total energy intake as the independent variable; and (b) the predicted nutrient intake at the mean energy intake [30]. All continuous variables were tested for normality using Kolmogorov-Smirnov and were all not normally distributed. Accordingly, Kruskal–Wallis and Mann–Whitney U tests were used to test for significance of group-differences. Chi-square test for independence or Fisher’s exact test were used for testing of association between categorical variables.

Nutrients intakes were extracted based on the Egyptian food composition table [20]. Principal Component Analysis (PCA) with Equimax rotation was performed on energy-adjusted intakes, and four factors (i.e., nutrient patterns) of an Eigen value ≥ 1 were extracted. Only factor loadings >0.30 were reported. A factor score was calculated for each extracted nutrient pattern and classified into tertiles. Given that all ADHD cases were in the third tertile of the first nutrient pattern, this pattern was excluded from the regression analysis. Binary logistic regression models were performed with ADHD status as the dependent variable and nutrient patterns’ tertiles as the independent variable (the crude model). The adjusted models were adjusted for child age, birth order, BMI percentiles, physical activity, screen time, family conflicts and violence, breast-feeding, mother education and work, low-birth weight, and Cesarian delivery. Odds ratios and 95% confidence intervals were calculated across the tertiles of the nutrient patterns, with *p* value for trend across the tertiles. Statistical significance was considered at *p* value < 0.05.

## Results

Children with ADHD and their community controls were significantly older than sibling (*p* < 0.001). Birth order was significantly different across the study groups, with 63% of ADHD cases being first-born, compared to 39% and 23.4% among community and sibling groups, respectively (*p* < 0.001). No significant differences in residence across groups. Children with ADHD had significantly higher mean BMI percentiles than their community controls and sibling (*p* < 0.001), while no significant different between community controls and siblings (*p* = 0.407). Further sociodemographic characteristics are presented in Table 1.

**Table 1:** Comparison of sociodemographic characteristics and BMI percentiles of studied children in the ADHD and control groups.

Characteristics	ADHD (*n* = 146)	Control groups			*p* value*s*	
		Community (*n* = 146)	Siblings (*n* = 141)	*p1*	*p2*	*p3*
Child gender						
Male	89 (61.0%)	89 (61.0%)	89 (63.1%)	0.910	–	–
Female	57 (39.0%)	57 (39.0%)	52 (36.9%)			
Child age (years), Mean ( ± SD)	7.7 ( ± 1.5)	7.8 ( ± 1.5)	6.7 ( ± 2.6)	<0.001*^k^	–	–
Birth order						
First	92 (63.0%)	57 (39.0%)	33 (23.4%)	<0.001*	<0.001*	<0.001*
Middle	36 (24.7%)	75 (51.4%)	53 (37.6%)			
Youngest	13 (8.9%)	14 (9.6%)	55 (39.0%)			
Residence						
Urban	91 (62.3%)	100 (68.5%)	90 (63.8%)	0.890	0.268	0.337
Rural	55 (37.7%)	46 (31.5%)	51 (36.2%)			
Mother’s Education						
Illiterate/read & write	12 (8.2%)	5 (3.4%)	12 (8.5%)	–	<0.001*	0.001*
Basic education	3 (2.1%)	16 (11.0%)	3 (2.1%)			
Secondary school	69 (47.3%)	48 (32.9%)	65 (46.1%)			
University and higher	62 (42.5%)	77 (52.7%)	61 (43.3%)			
Mother’s Work						
Housewife	85 (58.2%)	29 (19.9%)	83 (58.9%)	–	<0.001*	<0.001*
Manual work	39 (26.7%)	102 (69.9%)	36 (25.5%)			
Clerks/technician/sales	5 (3.4%)	3 (2.1%)	5 (3.5%)			
Professional job	17 (11.6%)	12 (8.2%)	17 (12.1%)			
BMI Percentiles						
Normal (5th–<85th)	78 (53.4%)	129 (88.4%)	133 (94.3%)	<0.001*	<0.001*	0.181
Overweight (85th–<95th)	53 (36.3%)	13 (8.9%)	6 (4.3%)			
Obese (≥95th)	15 (10.3%)	4 (2.7%)	2 (1.4%)			
Mean (SD)	70.16 (24.63)	51.69 (23.09)	49.70 (18.26)	<0.001*^m^	<0.001*^m^	0.407^m^
Physical Activity						
None	127 (87.0%)	28 (19.2%)	101 (71.6%)	<0.001*	<0.001*	<0.001*
Once per week or less	2 (1.4%)	79 (54.1%)	19 (13.5%)			
Twice or more per week	17 (11.6%)	39 (26.7%)	21 (14.9%)			
Screen time (>2 h daily)	125 (85.6%)	34 (23.3%)	132 (93.6%)	0.027*	<0.001*	<0.001*
Maternal age at childbirth (years) Mean ( ± SD)	27.29 ( ± 4.82)	27.53 ( ± 4.01)	28.24 ( ± 2.58)	0.058^m^	0.574^m^	0.251^m^
Cesarean delivery	97 (66.4%)	10 (6.8%)	53 (37.6%)	<0.001*	<0.001*	<0.001*
Low-birth weight (<2500 gm)	36 (24.7%)	1 (0.7%)	4 (2.8%)	<0.001*	<0.001*	0.208
Breast-feeding	109 (74.7%)	142 (97.3%)	128 (90.8%)	<0.001*	<0.001*	0.032*^f^
Family type:						
Nuclear family	127 (87.0)	122 (83.6)	124 (87.9)	–	0.690	0.546
Single-parent family	11 (7.5)	13 (8.9)	10 (7.1)			
Step/blend family	8 (5.5)	11 (7.5)	8 (5.5)			
Family Conflicts	33 (22.6%)	125 (85.6%)	30 (21.3%)	–	<0.001*	<0.001*
Family Violence	142 (97.3%)	85 (58.2%)	93 (66.0%)	<0.001*	<0.001*	0.177

*p1 p value* from comparing between ADHD group and Control II (sibling) group.

*p2 p value* from comparing between ADHD group and Control I (community) group.

*p3 p value* for community control vs sibling groups.

^*^Statistically significant *p* value at *p* < 0.05.

^f^Chi square or Fisher’s exact tests.

^k^Kruskal Wallis.

^m^Mann–Whitney Test.

In Table 2, total energy and energy-adjusted water, micro- and macronutrients intakes showed statistically significant variations across the study groups (*p* < 0.001). However, pairwise comparisons showed insignificant differences in energy-adjusted intakes of fats, copper, phosphorus, and vitamins A and C, when compared between community controls and siblings (*p* = 0.086, 0.081, 0.208, 0.084, and 0.813, respectively). ADHD group showed the highest daily total energy, and energy-adjusted intakes of carbohydrates and sodium, however, it showed the lowest daily energy-adjusted intakes of water and all other macro- and micronutrients.

**Table 2:** Daily macronutrients, water, energy, and energy-adjusted micronutrients intake among the ADHD group and control groups.

Type of intake	ADHD (*n* = 146)	Control groups		*p value*^a^	*Pairwise-adjusted p* value^b^		
		Community (*n* = 146)	Siblings (*n* = 141)		*p1*	*p2*	*p3*
*Total energy intake*, kJ (kcal) /d	8867.9 (2119.5)	4481.9 (1071.2)	7308.2 (1746.7)	<0.001*	<0.001*	<0.001*	<0.001*
Energy-adjusted^c^ Water (*ml/d*)	558.00	684.83	707.66	<0.001*	<0.001*	<0.001*	0.089
Energy-adjusted^d^ Macronutrients intake:							
*Carbohydrates (g/d)*	225.28	224.84	206.57	<0.001*	0.001*	0.987	<0.001*
*Proteins (g/d)*	56.76	60.58	64.52	<0.001*	<0.001*	<0.001*	<0.001*
*Fats (g/d)*	69.37	79.14	73.04	<0.001*	0.014*	<0.001*	0.086
Energy-adjusted^c^ Micronutrients:							
*Zinc* (*mg/d*)	6.67	7.16	7.60	<0.001*	<0.001*	<0.001*	<0.001*
*Calcium* (*mg/d*)	417.70	606.47	473.54	<0.001*	0.002*	<0.001*	<0.001*
*Copper* (*mg/d*)	0.63	0.76	0.81	<0.001*	<0.001*	<0.001*	0.081
*Iron* (*mg/d*)	9.51	10.07	10.79	<0.001*	<0.001*	0.011*	<0.001*
*Magnesium* (*mg/d*)	85.15	124.01	143.59	<0.001*	<0.001*	<0.001*	<0.001*
*Phosphorus* (*mg/d*)	674.28	773.04	752.09	<0.001*	<0.001*	<0.001*	0.208
*Potassium* (*mg/d*)	2083.83	2451.57	2570.98	<0.001*	<0.001*	<0.001*	0.003*
*Riboflavin* (*mg/d*)	0.58	0.86	0.82	<0.001*	<0.001*	<0.001*	0.002*
*Sodium* (*mg/d*)	1969.11	1843.47	1609.97	<0.001*	<0.001*	0.002*	<0.001*
*Thiamine* (*mg/d*)	0.49	0.59	0.72	<0.001*	<0.001*	<0.001*	<0.001*
*Vitamin A (µgRE)*	346.16	625.02	687.87	<0.001*	<0.001*	<0.001*	0.084
*Vitamin C* (*mg/d*)	50.04	76.24	85.51	<0.001*	<0.001*	<0.001*	0.813
*Fiber*	4.82	6.21	7.13	<0.001*	<0.001*	<0.001*	<0.001*

Daily intake is expressed as median.

*Statistically significant *p value* at *p* < 0.05.

^a^Kruskal–Wallis Test, and ^b^Mann–Whitney U test where:

*p1 p value* from comparison of the three groups ADHD group and sibling control group.

*p2 p value* from comparing between ADHD group and community control group.

*p3 p value* for community control vs sibling groups.

^c^Walter Willett’s method (i.e., Nutrient residual) for energy adjustment. All nutrients intakes were standardized to the predicted nutrient intake at the mean total energy intake in the study sample ( = 1750.15 kcal or 7322.6 KJ).

The PCA of the daily energy-adjusted nutrient intakes resulted in extraction of four factors (i.e., nutrient patterns) explaining 75.9% of the total variance. Table 3 shows the four extracted patterns named as: (1) Predominantly Calcium-Phosphorus minerals, (2) predominantly Vitamins, (3) Predominantly Zinc-Iron minerals, and (4) Macronutrient. Only factor loadings >0.30 were reported. First, nutrient pattern comprised mainly the energy-adjusted intakes of calcium and phosphorus, and riboflavin, while the second pattern involved a mixture of many minerals and vitamins. Third, nutrient pattern encompassed iron, zinc, and sodium intakes. Fourth, the pattern included the main macronutrients—carbohydrates, proteins, and fats.

**Table 3:** Factor loadings* for the major nutrients patterns derived from principal component analysis.

Energy-adjusted Nutrients	Nutrients Patterns (Factors)			
	Predominantly Minerals - Calcium-phosphorus	Predominantly Vitamins	Predominantly Minerals -Zinc-Iron	Macronutrients
Phosphorus	0.927			
Calcium	0.924			
Riboflavin	0.865			
Vitamin C		0.780		
Thiamine		0.714		
Water		0.689		
Copper		0.666		
Magnesium		0.637		
Fiber		0.629		
Potassium		0.532		
Vitamin A		0.442		
Iron			0.842	
Zinc			0.644	
Sodium			0.591	
Carbohydrates				0.924
Fats				−0.838
Proteins				−0.565

Extraction Method: Principal Component Analysis.

Rotation Method: Equamax with Kaiser Normalization.

*Only factor loadings with absolute values > 0.3 are listed in the table.

Table 4 compares the mean factor score of each nutrient pattern between all study groups. ADHD group had the least mean factor scores on the first three patterns, and the lowest mean score in the fourth nutrient pattern. In ADHD group, the mean factor scores of the first and second patterns were significantly lower than community controls (*p* < 0.001), while the second and third pattern’s factor scores were significantly lower than siblings (*p* < 0.001). The mean factor score of the fourth pattern (macronutrients) was significantly high in ADHD group when compared to either community controls or siblings (*p* < 0.001).

**Table 4:** Comparisons of factor scores of nutrient patterns in ADHD and control groups.

Nutrient Patterns	ADHD	Community Controls		Siblings	
	Mean (SD)	Mean (SD)	*p value* ^a^	Mean (SD)	*p value* ^a^
1st	−0.09 (1.47)	0.12 (0.45)	<0.001*	−0.03 (0.77)	0.277
2nd	−0.69 (0.82)	0.08 (0.63)	<0.001*	0.63 (1.04)	<0.001*
3rd	−0.11 (1.32)	−0.15 (0.55)	0.329	0.27 (0.92)	0.001*
4th	0.38 (0.99)	0.00 (0.45)	<0.001*	−0.39 (1.25)	<0.001*

1st: Predominantly- minerals (Calcium-phosphorus), 2nd: Predominantly- Vitamins, 3rd: Predominantly- minerals (Zinc-Iron), 4th: Macronutrients.

*Statistically Significant *p* values.

^a^Mann–Whitney U test.

In Table 5, binary logistic regression analysis was performed to test for the associations between the tertiles of each nutrient pattern’s score (independent variable) and the ADHD (dependent variable). Lower tertiles of the first two nutrient patterns were significantly associated with increasing odds of ADHD, when compared to community controls, and adjusted for study covariates, with significant dose-response gradient (*p* for trend = 0.002 and 0.005, respectively). Likewise, lower tertiles of the second and third nutrient patterns were significantly associated with increasing odds of ADHD, when compared to siblings, and adjusted for study covariates, with significant dose-response gradient (*p* for trend <0.001 and <0.001, respectively). However, higher tertiles of the fourth nutrient pattern was significantly associated with increasing odds of ADHD, when compared to either community controls or siblings, and adjusted for study covariates, with significant dose-response gradient (*p* for trend <0.001 and <0.001, respectively). Estimates of the odds ratios in the crude models and models adjusted for covariates only are presented in Table 5.

**Table 5:** Multivariate Regression for the association between the nutrients pattern scores and ADHD, compared to community children and sibling as controls.

Nutrient patterns (Factors)	Tertiles of factors scores	Column No. (%)			ADHD vs Community controls		ADHD vs Siblings	
		ADHD (*n* = 146)	Community controls (*n* = 146)	Siblings (*n* = 141)	Crude Model OR (95% CI)	Adjusted Model^a^ OR (95% CI)	Crude Model OR (95% CI)	Adjusted Model^a^ OR (95% CI)
1st	T1	60 (41.1%)	12 (8.2%)	67 (47.5%)	7.95 (3.80–16.63)	21.92 (3.37–142.34)	0.99 (0.57–1.72)	0.62 (0.22–1.75)
	T2	47 (32.2%)	72 (49.3%)	31 (22.0%)	1.04 (0.60–1.79)	1.84 (0.48–7.12)	1.67 (0.89–3.13)	1.53 (0.57–4.10)
	T3	39 (26.7%)	62 (42.5%)	43 (30.5%)	1	1	1	1
	*P* trend	<0.001*			<0.001*	0.002*	0.790	0.317
2nd	T1	94 (64.4%)	32 (21.9%)	18 (12.8%)	18.28 (8.13–41.09)	291.7 (38.86–2189.7)	45.84 (19.51–107.71)	156.05 (30.83–789.79)
	T2	43 (29.5%)	58 (39.7%)	44 (31.2%)	4.61 (2.06–10.34)	12.49 (2.57–60.67)	8.58 (3.83–19.24)	28.55 (6.59–123.64)
	T3	9 (6.2%)	56 (38.4%)	79 (56.0%)	1	1	1	1
	*P* trend	<0.001*			<0.001*	0.005*	<0.001*	<0.001*
3rd	T1	69 (47.3%)	46 (31.5%)	29 (20.6%)	1.18 (0.67–2.09)	4.95 (1.02–24.19)	3.04 (1.70–5.41)	17.67 (4.75–65.68)
	T2	30 (20.5%)	63 (43.2%)	52 (36.9%)	0.38 (0.20–0.69)	10.06 (1.77–57.20)	0.74 (0.41–1.33)	4.01 (1.21–13.29)
	T3	47 (32.2%)	37 (25.3%)	60 (42.6%)	1	1	1	1
	*P* trend	0.351			0.353	0.103	<0.001*	<0.001*
4th	T1	35 (24.0%)	38 (26.0%)	71 (50.4%)	1	1	1	1
	T2	35 (24.0%)	76 (52.1%)	34 (24.1%)	0.50 (0.27–0.92)	1.23 (0.26–5.75)	2.09 (1.12–3.89)	2.51 (0.93–6.74)
	T3	76 (52.1%)	32 (21.9%)	36 (25.5%)	2.58 (1.39–4.78)	5.60 (1.15–27.18)	4.28 (2.43–7.55)	8.13 (2.91–22.71)
	*P* trend	<0.001*			<0.001*	0.017*	<0.001*	<0.001*

*T* tertile, where T1 is the lowest intake and T3 is the highest intake.

1st: Predominantly- Minerals (Calcium-phosphorus), 2nd: Predominantly- Vitamins, 3rd: Predominantly- Minerals (Zinc-Iron), 4th: Macronutrients.

*Statistically Significant *p* value at *p* < 0.05.

^a^Adjusted for child age, birth order, BMI percentiles, physical activity, screen time, family conflicts and violence, breast-feeding, mother education and work, low-birth weight, Cesarian delivery.

## Discussion

This study revealed that ADHD children had significantly higher daily total energy, and energy-adjusted intakes of carbohydrates and sodium, compared to controls. However, they had significantly low energy-adjusted daily intake of water and all other micro- and macronutrients. The study also identified four nutrient patterns among children: predominantly minerals (Calcium-Phosphorus), predominantly-vitamins, predominantly minerals (Zinc-Iron) and macronutrients. Most of the recruited children in the three groups were males, firstborns, and living in urban areas with nuclear families. These characteristics were comparable to those described in several studies investigating children diagnosed with ADHD [7, 31–33].

ADHD children in our study showed a low energy-adjusted daily intake of all observed micronutrients except for sodium whose intake was high. This is most probably sodium benzoate because of frequent consumption of certain preserved food products as stock, potato chips, pickles, and tomato paste, where the ADHD cases had higher weekly or even daily consumption. In this respect, attention is being paid to sodium benzoate consumption, which was strongly associated with ADHD. Moreover, increased salt intake was associated with a higher risk of ADHD in children [34].

Low intake of the rest of the observed micronutrients among ADHD cases is in line with previous studies that linked low intake of these micronutrients with the increased risk of ADHD. On the other hand, the carbohydrate intakes were increased among our ADHD cases, while fat intake was reduced, which is consistent with previous research as well [35–37].

The linkage between macro- and micronutrient to ADHD in children could be explained by nutrient deficiencies due to attentional demands and medication effects. ADHD is caused by neurotransmitters dopamine and norepinephrine, which are influenced by iron, zinc, and copper. Low zinc levels in DHD patients affect attention scores but not hyperactivity, while low serum ferritin is linked to severe ADHD symptoms [18, 35–39].

The first nutrient pattern, named as “predominately-minerals/Calcium-Phosphorus” comprised calcium, riboflavin, and phosphorus. Its intake was higher among the sibling group, and its increased intake was associated with lower odds of ADHD. This finding is in line with previous research signaling the vital role of these elements in protecting against ADHD [38, 40–43].

The second nutrient pattern, named “predominantly-vitamins” comprised vitamin C, fibers, copper, vitamin A, magnesium, and thiamine. The mean intake of this pattern was higher among both control groups, and its increased intake was associated with lower ADHD odds. This would suggest a possible role of this pattern in protecting against ADHD. In concordance with previous research, all the nutrients included in this pattern were suggested to play a major role in protecting against ADHD [11, 12, 38, 43, 44].

Conversely, Chou et al. [41] did not find any significant difference regarding calcium intake between ADHD cases and controls in their study. This contradiction between our results and theirs could be attributed mainly to the big difference in the number of the food items investigated, where our study inquired about 116 food items, while their study investigated 49 food items only. Another variation between the two studies is the age range, where Chou et al. [41] recruited cases 6–16 years old, while our cases were 6–10 years old.

An important strength in our study is that we recruited newly diagnosed cases of ADHD, and we inquired about their dietary habits in the preceding year. This might help to some extent in proposing a temporal relationship between dietary patterns as an environmental risk factors and ADHD. Nevertheless, several limitations should be considered while interpreting our findings. First, we could not recruit all the siblings of the cases with a maximum age difference of 2 years, where some siblings were older by 3 years; this led to a sibling control group composed of children and some adolescents. This would probably impact on the amount of food intake and food preferences. Second, recall bias should be considered since we had inquired about the dietary data from a previous year. Third, although our study investigated most of the possible cofactors prevalent in the target population, the total variance explained by the nutrient patterns was 75.9%, thus indicating other essential factors influencing ADHD. Nonetheless, we assume that the remaining variance is explained by genetic susceptibility that is then triggered by the dietary patterns consumed by the children. Lastly, the study design (case-control design) could not establish the causal relationship between nutrients patterns and ADHD.

Furthermore, our study adapted the latest Egyptian food composition tables that were released in 2006. This led to some challenges, such as the absence of some food items investigated in our study from the food composition table. To solve this problem, we used nutritionally similar food items that are present in the food composition table to be surrogates. Last, some food products that are strongly associated with ADHD, could not be quantitatively investigated in our study due to the difficult accurate estimation of the amount delivered to the child. For example, the consumption of trans fat is strongly associated with ADHD; however, our study inquired about whether the parents use it during cooking and thus could not estimate the exact quantity delivered to the child.

This study concluded that lower intakes of nutrients patterns of predominantly minerals and vitamins, and the higher intakes of macronutrients were associated with increased likelihood of ADHD in children. These findings can inform parents, caregivers, policy- and decision-makers, media, and food industry about the importance of healthy food consumption and good-quality dietary intake in early prevention of ADHD. Further prospective studies are needed to ascertain the causal relationship between nutrient patterns and ADHD development, while randomized control trials are needed with focus on caregiver education and tailored nutritional regimens for improved mental health.

### Supplementary information

PCA for dietary patterns

## Data Availability

The data that support the findings of this study are available from the authors upon reasonable request.
