# Supplementary material for: Nutrients patterns and attention deficit hyperactivity disorder among Egyptian children: a sibling and community matched case-control study
Source: Eur J Clin Nutr. 2023 Sep 26;78(1):64–71. doi: 10.1038/s41430-023-01345-0 (PMC10774123; doi:10.1038/s41430-023-01345-0)
Supplement: Supplementary file 1 — PCA for dietary patterns [file 41430_2023_1345_MOESM1_ESM.docx]

**Supplementary Table (S1): PCA for dietary pattens**


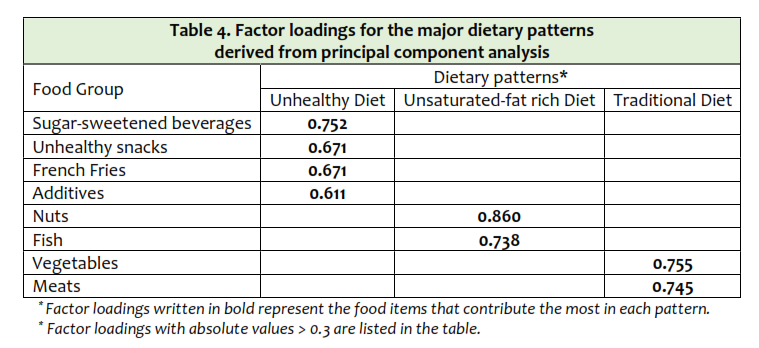


**Supplementary Table (S2): Factors scores of dietary patterns by the study groups.**

**
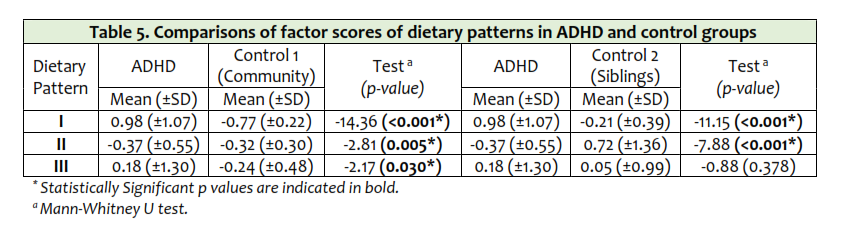
**
